# A Highly Warped Heptagon‐Containing sp^2^ Carbon Scaffold via Vinylnaphthyl π‐Extension

**DOI:** 10.1002/anie.201909975

**Published:** 2019-09-30

**Authors:** Jeffrey M. Farrell, Vincenzo Grande, David Schmidt, Frank Würthner

**Affiliations:** ^1^ Institut für Organische Chemie Universität Würzburg Am Hubland 97074 Würzburg Germany; ^2^ Center for Nanosystems Chemistry (CNC) Universität Würzburg Theodor-Boveri-Weg 97074 Würzburg Germany

**Keywords:** arenes, carbon, C−C coupling, curvature, polycyclic aromatic hydrocarbons

## Abstract

A new strategy is demonstrated for the synthesis of warped, negatively curved, all‐sp^2^‐carbon π‐scaffolds. Multifold C−C coupling reactions are used to transform a polyaromatic borinic acid into a saddle‐shaped polyaromatic hydrocarbon (**2**) bearing two heptagonal rings. Notably, this Schwarzite substructure is synthesized in only two steps from an unfunctionalized alkene. A highly warped structure of **2** was revealed by X‐ray crystallographic studies and pronounced flexibility of this π‐scaffold was ascertained by experimental and computational studies. Compound **2** exhibits excellent solubility, visible range absorption and fluorescence, and readily undergoes two reversible one‐electron oxidations at mild potentials.

## Graphical abstract

**Carbonaceous and curvaceous**: A new strategy is described for the synthesis of heptagon‐containing all‐sp^2^‐hybridized carbon scaffolds from unfunctionalized alkene precursors. This direct approach is applied for the synthesis of a flexible, negatively curved polyaromatic hydrocarbon that exhibits excellent solubility, visible range absorption and fluorescence, and two reversible one‐electron oxidations at mild potentials.

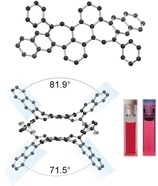

Extended 2D frameworks of sp^2^‐hybridized carbon continue to provoke scientific attention for their fascinating chemical, electronic, magnetic and photophysical properties.^1^ Diverse structures such as graphene, polycyclic aromatic hydrocarbon (PAH) “nanographenes,” carbon nanotubes, and fullerenes have had a revolutionary impact on diverse fields such as photovoltaics, host‐guest chemistry, and organic electronics. In recent research, inclusion of curvature has been identified as an intriguing way to manipulate electronics and to impart dynamic structural behavior in sp^2^ carbon frameworks.^2^ Curved compounds often show improved solubility and fluorescence properties compared to their planar counterparts. Moreover, these compounds are promising candidates to mediate multi‐dimensional charge transport in the solid state. In order to synthesize curved polyaromatics, non‐six‐membered rings are included in polybenzenoid frameworks. Positive curvature, as seen in fullerenes or corannulenes, is typically obtained by the inclusion of five‐membered rings. Conversely, negative curvature, present in rarer saddle‐shaped structures, is generally achieved by the inclusion of heptagonal or octagonal rings.^3^

With elegant structures and intriguing properties, extended negatively curved sp^2^ carbon structures have become the focus of intense recent study.^2^, ^4^ Nevertheless, synthetic strategies for negatively curved PAHs bearing heptagonal rings remain relatively few. Certainly, warping and ring‐strains that destabilize final products pose synthetic barriers. Furthermore, strategies that provide “pristine” all‐sp^2^, carbon‐only π‐scaffolds present additional challenges. These stipulations preclude solubility‐enhancing substituents and heteroatom directing groups to aid C−C bond forming reactions. These latter targets are especially appealing, however, as they can be viewed as subunits of new carbon allotropes extending in three dimensions (i.e., “Schwarzites”, or “Mackay crystals”).^5^, ^6^

Synthetic strategies for curved, heptagon‐containing sp^2^ carbon structures most often utilize intramolecular oxidative C−H/C−H couplings^7^ or Pd‐catalyzed C−H/C−X couplings^8^ of prearranged aromatic units as key steps. Other recent approaches involve heptagonal ketone precursors,^9^ which can in turn be made by cyclotrimerization^10^ or ring expansion.^11^ Intermolecular coupling routes are less commonly encountered, but are promising in terms of generalizing reactivity.^12^

Recently our group has reported a facile one‐pot synthetic approach for PAHs bearing embedded borinic acid moieties using simple alkene precursors.^13^ This method gives access to previously challenging polyaromatic borinic acids that in turn allows their practical study in further roles. Specifically, we envisioned that polyaromatic borinic acids could be used as intermolecular coupling partners with *o*‐dihaloarenes where the prearrangement of borinic acid C−B bonds would lead to seven‐membered carbocycles (Scheme 1). In this way, pristine all‐sp^2^, carbon‐only π‐scaffolds bearing heptagonal rings would be accessible in only two steps from alkenes. Herein we validate this approach with the synthesis of a highly warped PAH bearing two seven‐membered rings. The compound was studied by X‐ray crystallography, DFT calculations, UV/Vis spectroscopy, fluorescence spectroscopy and cyclic voltammetry. These studies reveal a highly warped, but flexible, carbon π‐scaffold that represents a substructure of a previously predicted Schwarzite. The compound also exhibits excellent solubility, facile electrochemical oxidations and visible range absorption and fluorescence.

**Scheme 1:**
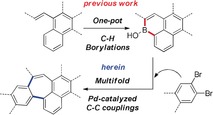
Synthetic strategy devised for the construction of heptagon‐containing carbon π‐scaffolds.

We began our study by screening reaction conditions for the four‐fold Pd‐catalyzed C−C coupling reaction between borinic acid **1** and 2,3‐dibromonaphthalene (see Supporting Information, Table S1). Initial conditions were based on those shown for the two‐fold C−C coupling of dibromoaromatics with dibenzoxaborininols by Taylor and co‐workers.^14^ Despite structural and electronic differences between these substrates and ours, we found similar optimal conditions using Pd_2_dba_3_ and [*t*‐Bu_3_PH][BF_4_] as catalyst precursors and *t*‐amyl alcohol as a solvent (Table S1). The desired product was not observed when other screened solvents or ligands were employed, but the addition of small quantities of water improved yields. More concentrated reaction conditions did not improve yields. Optimized conditions afforded the desired polyaromatic **2** as a dark red solid in 41 % yield after column chromatography (Scheme 2). Compound **2** is bench stable as a solid, showing no signs of decomposition by NMR spectroscopy after storage in ambient conditions for months. However, signs of decomposition are observed by ^1^H NMR spectroscopy within days if **2** is kept in aerobic solution under ambient light. As noted for other warped PAHs, compound **2** shows excellent solubility in halogenated and in most non‐halogenated organic solvents. Interestingly, the connectivity of the all‐sp^2^ carbon framework of **2** is identical to an 11‐ring section of a stable Schwarzite (6‐1‐2‐p) predicted by Kotani and co‐workers (Figure 1).^15^

**Figure 1:**
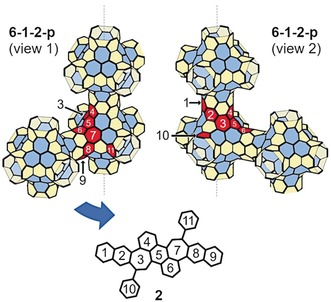
Comparison of the ring structure of **2** with the structure of Schwarzite 6‐1‐2‐p predicted by Kotani and co‐workers.^15^ A section of the Schwarzite is highlighted in red where the connectivity of six‐ and seven‐membered rings of **2** corresponds to analogous connectivity in the Schwarzite structure. To visualize the entirety of this overlap, two views of the section are given based on rotation about the axis labelled with a dotted line.

**Scheme 2:**
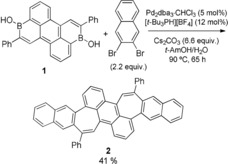
Synthesis of heptagon‐containing PAH **2** by Pd‐catalyzed C−C coupling.

Crystals suitable for X‐ray crystallography could be obtained by the slow evaporation of 1:1 diethyl ether/acetonitrile solutions of **2**. Analysis of structural data reveals a highly warped *cisoid* saddle‐shaped structure of **2** in the solid state with annulated naphthyl groups situated on the same side of the central anthracene unit (Figure 2). Planes described by the coupled naphthyl groups intersect at an 81.9° or a 71.5° angle in each molecule of the asymmetric unit (Figure 2 c). In its observed *cisoid* conformation, **2** is inherently chiral and possesses a C2 symmetry axis. The achiral *transoid* S‐shaped conformation (with an inversion center) is not observed in the solid‐state structure. Individual molecules of **2** pack as dimers with π‐systems separated by 3.8 Å at their closest point. In addition, close C−H/π contacts of ≈2.8 Å are observed between hydrogens of the naphthyl moieties of molecules of **2** and the π‐surfaces of neighboring molecules (Figure 2 d). The core anthracene fragment of **2** is significantly distorted from planarity with central benzene C−C bond torsion angles as high as 20.5°. The bonds between C1, C2, C3 and C4 in **2** are 1.462(3) Å, 1.348(3) Å and 1.470(2) Å, respectively (Figure 2 b). In comparison, the reported structure of 9,10‐distyrylanthracene (the alkene precursor to **2**) has bond lengths of 1.488(4) Å, 1.297(4) Å, and 1.475(4) Å at analogous positions.^16^ The lesser alternation of bond lengths in **2** compared to this precursor indicates a greater degree of electron delocalization, although the C2−C3 bond of **2** apparently retains considerable double‐bond character.

**Figure 2:**
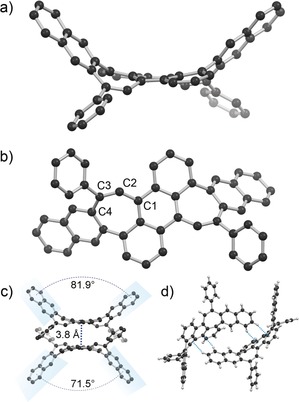
a) Side‐on and b) top‐down views of the solid‐state structure of **2** (H atoms omitted for clarity), c) a π‐stacked dimer of **2** (H atoms omitted for clarity) and d) two molecules of **2** with close C−H/π contacts illustrated by dotted blue lines. C: black, H: grey, solvent omitted.

The *cisoid* structure of **2** determined from X‐ray crystallography was further probed with DFT calculations at the B3LYP/6‐31+G* level of theory. The calculated geometry‐optimized structure agreed well with the structure obtained from X‐ray crystallography. Curved π‐systems often exhibit dynamic structural behavior and may have more than one stable conformer. Therefore, calculated electronic energies were used to estimate the relationships of various conformers of **2**. The observed saddle‐shaped *cisoid* structure of **2** was calculated to be 15 kJ mol^−1^ lower in energy than the alternative *transoid* conformation (see Supporting Information, Figure S7). The energetic barrier between these two species was calculated to be 42 kJ mol^−1^, which is fairly low amongst energetic barriers reported for similar conformation changes in other heptagon‐containing carbon π‐scaffolds.3b, 4 This low energetic barrier implies facile interconversion between the two conformations in solutions at room temperature with the energetically favored saddle‐shaped *cisoid* conformation predominating (*K*_eq_≈430). Therefore, although racemic *cisoid* **2** predominates in solution, its enantiomers rapidly interconvert via an achiral *transoid* intermediate. This calculated assessment of conformational behavior was in line with variable‐temperature ^1^H NMR spectroscopy in CD_2_Cl_2_, where peaks corresponding to a second conformer were not observed at as low as −90 °C (see Supporting Information, Figure S3).^17^

Compound **2** absorbs in the visible range in chloroform solution with *λ*_abs_=533 nm (1.68×10^−5^ m, 298 K) and an extinction coefficient of 12 900 L mol^−1^ cm^−1^ (Figure 3 a). Emission in chloroform solution occurs in the visible range with *λ*_em_=630 nm (5.44×10^−6^ m in CHCl_3_, 298 K) but extends into the NIR. These data correspond to a large Stokes shift of 2890 cm^−1^. The fluorescence quantum yield was measured as *Φ*=0.41 with a lifetime of 3.2 ns. In line with its more extensive conjugation, the absorption and fluorescence spectra of **2** are bathochromically shifted with respect to 9,10‐distyrylanthracene.^18^ Indeed, DFT calculations at the B3LYP/6‐31+G* level of theory suggest the delocalization of the frontier molecular orbitals over the seven‐membered rings of **2**, including the edges of the annulated naphthyl groups (Figure 3 b). Our spectroscopic studies of **2** suggest excited‐state non‐radiative energy loss that may be associated with the remarkable flexibility of this chromophore, somewhat resembling acene‐annulated octatetraenes introduced by Saito, Yamaguchi and co‐workers.^19^

**Figure 3:**
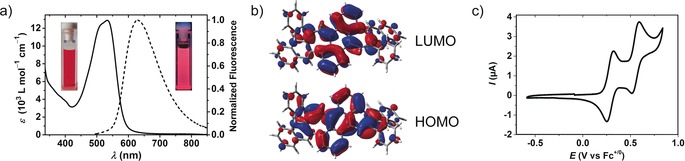
a) UV/Vis absorption spectrum (1.68×10^−5^ m in CHCl_3_, 298 K, solid line) and fluorescence spectrum (5.44×10^−6^ m in CHCl_3_, *λ*_exc_=490 nm, 298 K, dashed line) of **2**. Inset: Photographs of a solution of **2** in ambient light (left) and under 366 nm UV illumination (right). b) Frontier molecular orbitals of **2** from DFT calculations at the B3LYP/6‐31+G* level of theory. c) Cyclic voltammogram of **2** (2.5×10^−4^ m, 0.1 m *n*‐Bu_4_NPF_6_ in CH_2_Cl_2_, 298 K).

With prospective applications for curved aromatics in charge transport applications, we probed the electrochemical properties of **2** by cyclic voltammetry. We anticipated facile electrochemical oxidations of **2** due to the presence of heptagonal rings, which presumably exhibit tropylium‐like aromaticity upon electron loss. Indeed, two reversible one‐electron oxidations of **2** were observed by cyclic voltammetry in CH_2_Cl_2_ solution (2.5×10^−4^ m, 0.1 m *n*‐Bu_4_NPF_6_, 298 K). The oxidations occur at mild potentials of 0.29 V and 0.56 V vs. the ferrocenium/ferrocene (Fc^+/0^) redox couple (Figure 3 c). These are considerably lower potentials than even the first oxidation of 9,10‐distyrylanthracene, which occurs at 0.76 V vs. Fc^+/0^.^20^

In conclusion, we have devised a new synthetic approach toward all‐sp^2^‐carbon scaffolds containing seven‐membered rings. Utilizing multi‐fold Pd‐catalyzed C−C coupling of a polyaromatic borinic acid, we have synthesized an extended, highly warped polycyclic sp^2^‐hybridized carbon molecule (**2**) bearing two heptagonal rings that represents an 11‐ring substructure of a previously predicted Schwarzite. Our method represents a new and facile approach for the introduction of negative curvature into sp^2^ carbon scaffolds in only two synthetic steps from unfunctionalized alkenes. This provides an addition to other important π‐extension reactions of unfunctionalized hydrocarbons such as bay‐ or K‐region annulations.^21^ X‐ray crystallography and DFT studies reveal that compound **2** is highly warped and adopts a *cisoid* saddle‐shaped structure with planes of rings on either side of the π‐scaffold intersecting at angles less than 82°. DFT calculations and NMR spectroscopic studies suggest high flexibility of the scaffold. Compound **2** is highly soluble, exhibits visible range absorption and emission in solution, and is reversibly oxidized at mild potentials. We anticipate that this new, straightforward way to incorporate heptagonal rings into sp^2^‐hybridized carbon frameworks will offer access to other exciting new structures and studies of these are underway in our laboratories.

## Conflict of interest

The authors declare no conflict of interest.

## Supporting information

As a service to our authors and readers, this journal provides supporting information supplied by the authors. Such materials are peer reviewed and may be re‐organized for online delivery, but are not copy‐edited or typeset. Technical support issues arising from supporting information (other than missing files) should be addressed to the authors.

Supplementary
