# Supplementary material for: A Highly Warped Heptagon‐Containing sp2 Carbon Scaffold via Vinylnaphthyl π‐Extension
Source: Angew Chem Int Ed Engl. 2019 Sep 30;58(46):16504–7. doi: 10.1002/anie.201909975 (PMC6900032; doi:10.1002/anie.201909975)
Supplement: Supplementary file 1 — Supplementary [file ANIE-58-16504-s001.pdf]

Angewandte Chemie
Eine Zeitschrift der Gesellschaft Deutscher Chemiker

Supporting Information

A Highly Warped Heptagon-Containing sp^2 Carbon Scaffold via Vinylnaphthyl π-Extension

*Jeffrey M. Farrell, Vincenzo Grande, David Schmidt, and Frank Würthner**

anie_201909975_sm_miscellaneous_information.pdf

Table of Contents

1) Materials and Methods……S2
2) Synthetic Procedures …… S3
3) NMR Spectra…… S5
4) X-ray Crystallography……S8
5) Computations…… S9
6) References……S14

1) Materials and Methods

General Considerations: 2,8-Diphenyl-3,9-dihydroxy-3,9-diboraperylene (**1**)[S1] and $Pd_2dba_3{\cdot}CHCl_3$[S2] were prepared according to literature. 2,3-Dibromonaphthalene was obtained from Apollo Scientific (UK) and used without further purification. Tri-*t*-butylphosphonium tetrafluoroborate was obtained from Fluorochem (UK) and used without further purification. *t*-Amyl alcohol was obtained from Merck (DE) and used without further purification. Cs_2CO_3 was obtained from TCI (JP) and used without further purification. Anhydrous dimethylacetamide and toluene were from Sigma Aldrich. Chloroform for spectroscopic measurements was spectroscopic grade and was degassed by purging with nitrogen before use. Column chromatography was performed with commercial glass columns using silica gel 60M (particle size 0.04-0.063 mm). All other reagents and solvents were obtained from commercial sources and used without further purification.

UV-Vis absorption spectra were recorded on a Jasco V-670 spectrophotometer.

Fluorescence spectra were recorded on an Edinburgh Instruments FLS980 fluorescence spectrometer. The relative fluorescence quantum yield was measured in chloroform using the comparative method at four excitation wavelengths (484 nm, 486 nm, 488 nm, and 490 nm) with *N*,*N'*-bis(2,6-diisopropylphenyl)-1,6,7,12-tetraphenoxy-3,4:9,10-perylenebis(dicarboximide) in $CHCl_3$[S3] as a standard. Time-resolved measurements were performed with an Edinburgh Instruments picosecond pulsed laser diode (479.9 nm) and a TCSPC detection unit.

NMR spectra were recorded on Bruker Avance III HD 400 or Bruker Avance III HD 600 spectrometers. Chemical shifts are listed in parts per million given relative to $SiMe_4$ and are referenced to a residual solvent signal (^1H, ^{13}C). Coupling constants (*J*) are quoted in Hertz (Hz).

High resolution mass spectrometry was carried out on a Bruker Daltonics micrOTOF focus instrument.

Cyclic voltammetry was carried out using a standard commercial electrochemical analyzer (EC epsilon; BAS Instruments, UK) with a three-electrode single-compartment cell. The supporting electrolyte tetrabutylammonium hexafluorophosphate (n-Bu_4NPF_6) was prepared according to literature,[S4] and recrystallized from ethanol/water. The measurements were carried out using ferrocene (Fc) as an internal standard for the calibration of the potential. An Ag/AgCl reference electrode was used. A Pt disc and a Pt wire were used as working and auxiliary electrodes, respectively.

Single Crystal X-ray diffraction data were collected at 100 K on a Bruker D8 Quest Kappa diffractometer with a Photon100 CMOS detector and multi-layered mirror monochromated CuK_α radiation. The structures were solved using direct methods, expanded with Fourier techniques and refined with the Shelx software package.[S5] All non-hydrogen

atoms were refined anisotropically. Hydrogen atoms were included in the structure factor calculation on geometrically idealized positions. Crystallographic data have been deposited with the Cambridge Crystallographic Data Centre as supplementary publication no. CCDC 1945356 (**2**). These data can be obtained free of charge from The Cambridge Crystallographic Data Centre via www.ccdc.ac.uk/data.request/cif.

Computational details

Geometry optimizations were performed at the level of density functional theory (DFT) employing B3LYP as functional[S6-8] and 6-31+G* as basis set[S9-11] as implemented in the Gaussian 09 program package.[S12] The geometries were optimized followed by frequency calculations to confirm the existence of minima. The energetic barrier between cisoid and transoid conformations of **2** was estimated by calculating the energies of different geometries with varying torsion angles *α* and *β* through which **2** presumably traverses between conformations (see Figure S7).

2) Synthetic Procedures

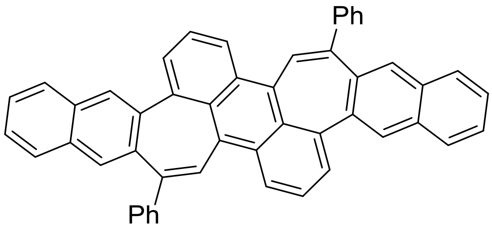

2

Synthesis of 2: 2,8-Diphenyl-3,9-dihydroxy-3,9-diboraperylene (**1**) (39.1 mg, 0.0900 mmol, 1 equiv.), 2,3-dibromonapthalene (56.6 mg, 0.198 mmol, 2.2 equiv.), Cs_2CO_3 (194 mg, 0.594 mmol, 6.6 equiv.), [*t*-Bu_3PH][BF_4] (3.1 mg, 0.011 mmol, 12 mol%) and $Pd_2dba_3{\cdot}CHCl_3$ (4.7 mg, 0.0045 mmol, 5 mol%) were weighed into a 50 mL Schlenk flask equipped with a magnetic stir bar. The vessel was sealed with a rubber septum, evacuated, and backfilled with nitrogen gas. *t*-AmOH (24 mL) was added via syringe and the reaction mixture was purged with argon. After 20 minutes, deionized water (72 mg) was added to the reaction mixture. After an additional five minutes, the vessel was equipped with an argon balloon, bubbling was ceased, and the reaction was stirred at room temperature for one hour. The reaction was then stirred at 90 °C for 65 hours and cooled to room temperature. The reaction mixture was poured into 1L EtOAc, filtered through celite then concentrated *in vacuo*. Following purification by column chromatography (19:1 hexane/EtOAc, R_f = 0.22) and concentration *in vacuo*, **2** could be isolated as a deep red solid (23.0 mg, 0.0365 mmol, 41 % yield). Crystal suitable for X-ray crystallography could be obtained by slow evaporation of a solution of **2** in 1:1 Et_2O/CH_3CN. **^{1}H NMR** (400 MHz, CD_2Cl_2, 295 K): δ 8.42 (dd, 2H, $^3J_{HH}$ = 8.7 Hz, $^4J_{HH}$ = 1.1 Hz), 7.83 (dd, 2H, $^3J_{HH}$ = 7.1 Hz, $^4J_{HH}$ = 1.0 Hz), 7.81-7.77 (m, 2H), 7.75-7.71 (overlapping m, 6H), 7.70-7.66 (m, 2H), 7.61 (br, overlapping s, 4H), 7.53-7.47 (m, 4H), 7.46-7.39 (overlapping m and s, 8H). **^{13}C{^{1}H} NMR** (101 MHz, CD_2Cl_2, 295 K): δ 145.0 (2C, *C*), 143.1 (2C, *C*), 141.5 (2C, *C*), 138.4 (2C, *C*), 137.4 (2C, *C*), 136.5 (2C, *C*), 134.8 (2C, *C*), 132.9 (2C, *C*H), 132.7 (2C, *C*H), 132.6 (2C, *C*), 130.71 (2C, *C*), 129.6 (4C, CH), 129.5 (2C, *C*H), 129.2 (2C, *C*), 129.0 (4C, CH), 128.8 (2C, *C*H), 128.12 (2C, *C*H), 128.05 (2C, *C*H), 128.03 (2C, *C*H), 127.5 (2C, *C*H), 126.9 (2C, *C*H), 126.7 (2C, *C*H), 124.3 (2C, *C*H). **MP**: > 250 °C. **HR-MS** (ESI TOF, positive mode) *m/z*: [M]$^+$ Calc'd for $C_{50}H_{30}$ 630.2342; Found 630.2345. **CV** (2.5 x 10^{-4} M, 0.1 M *n*-Bu_4NPF_6

in CH_2Cl_2 295 K): $E_{1/2\,ox\,1}$ = 0.29 V, $E_{1/2\,ox\,2}$ = 0.56 V. **UV-Vis**: (1.68 x 10^{-5} M, $CHCl_3$, 295 K: λ_{max} (ε_{max}) = 533 nm (12 900 L mol^{-1} cm^{-1}). **Fluorescence** (5.44 x 10^{-6} M, $CHCl_3$, λ_{exc} = 490 nm, 295 K): λ_{max} = 630 nm (Φ = 0.41).

Optimized conditions were arrived at through screening solvent, solvent volume, and ligand/ precursor as shown in table S1. All other reaction conditions were identical to those described above.

Table S1. Optimization of Multi-fold Palladium-Catalyzed C—C coupling for Heptagon-Containing PAH 2.

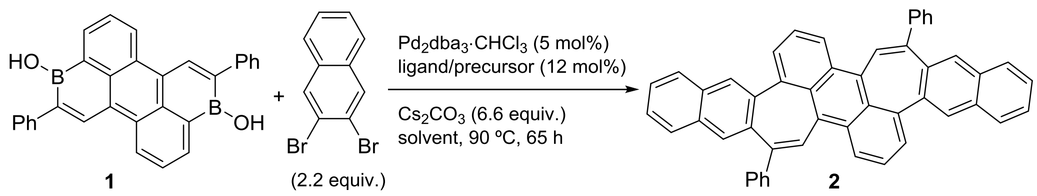

entry	ligand/precursor	solvent	% yield[a]
1	P(*o*-tolyl)$_3$	*t*-AmOH	0
2	IdippHCl	*t*-AmOH	0
3	HBF_4·PCy_3	*t*-AmOH	0
4	HBF_4·P(*t*-Bu)$_3$	*t*-AmOH	9
5	HBF_4·P(*t*-Bu)$_3$	toluene	0
6	HBF_4·P(*t*-Bu)$_3$	DMAc	0
7[b]	HBF_4·P(*t*-Bu)$_3$	*t*-AmOH	41
8[b, c]	HBF_4·P(*t*-Bu)$_3$	*t*-AmOH	26

[a] Isolated Yields. [b] 72 mg water added to reaction mixture. All reactions carried out in 24 mL solvent except [c] carried out in 6 mL solvent. IdippHCl: 1,3-bis(2,6-diisopropylphenyl)imidazolium chloride.

3) NMR Spectra

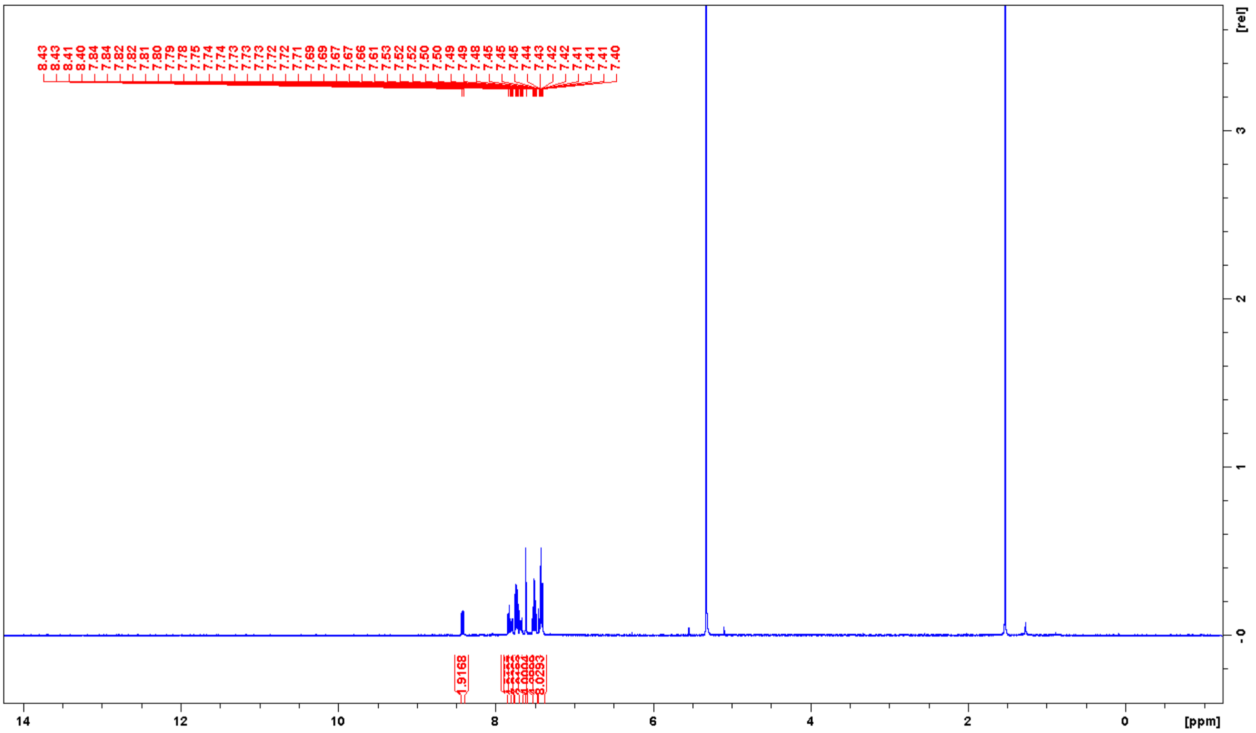

Figure S1. ^{1}H NMR spectrum of **2** (400 MHz, CD_2Cl_2, 295 K).

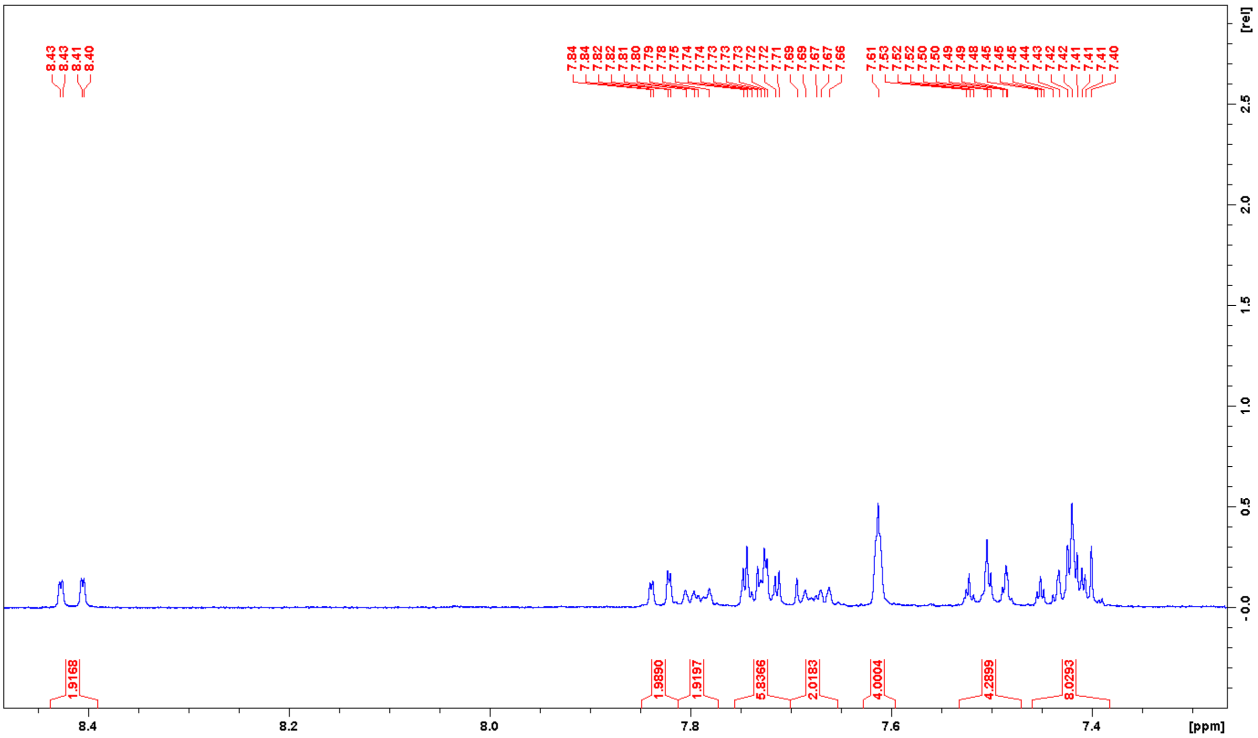

Figure S2. Magnified aromatic region of the ^{1}H NMR spectrum of **2** (400 MHz, CD_2Cl_2, 295 K).

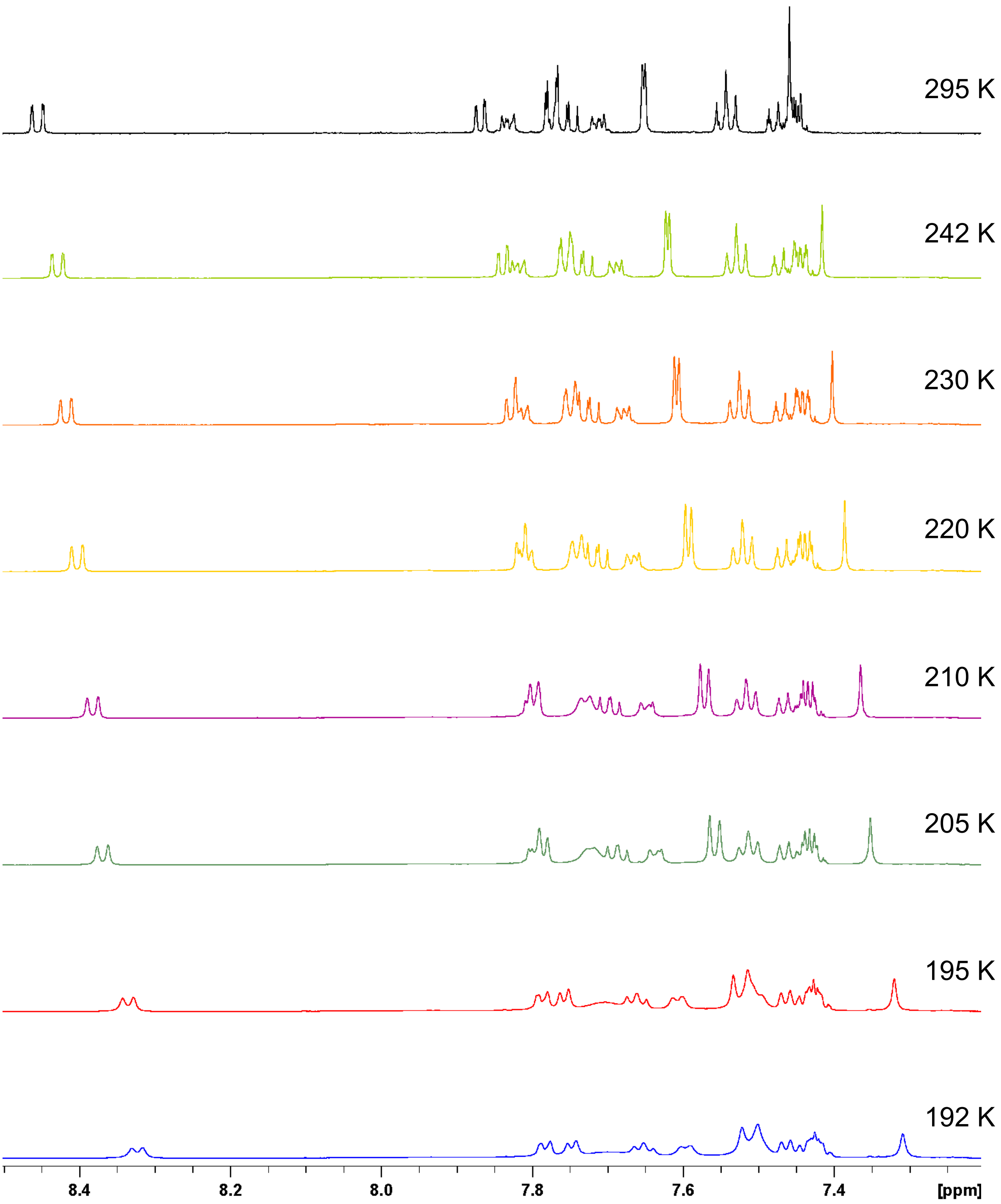

Figure S3. Magnified aromatic regions of the ^{1}H NMR spectra of **2** collected at variable temperatures (600 MHz, CD_2Cl_2).

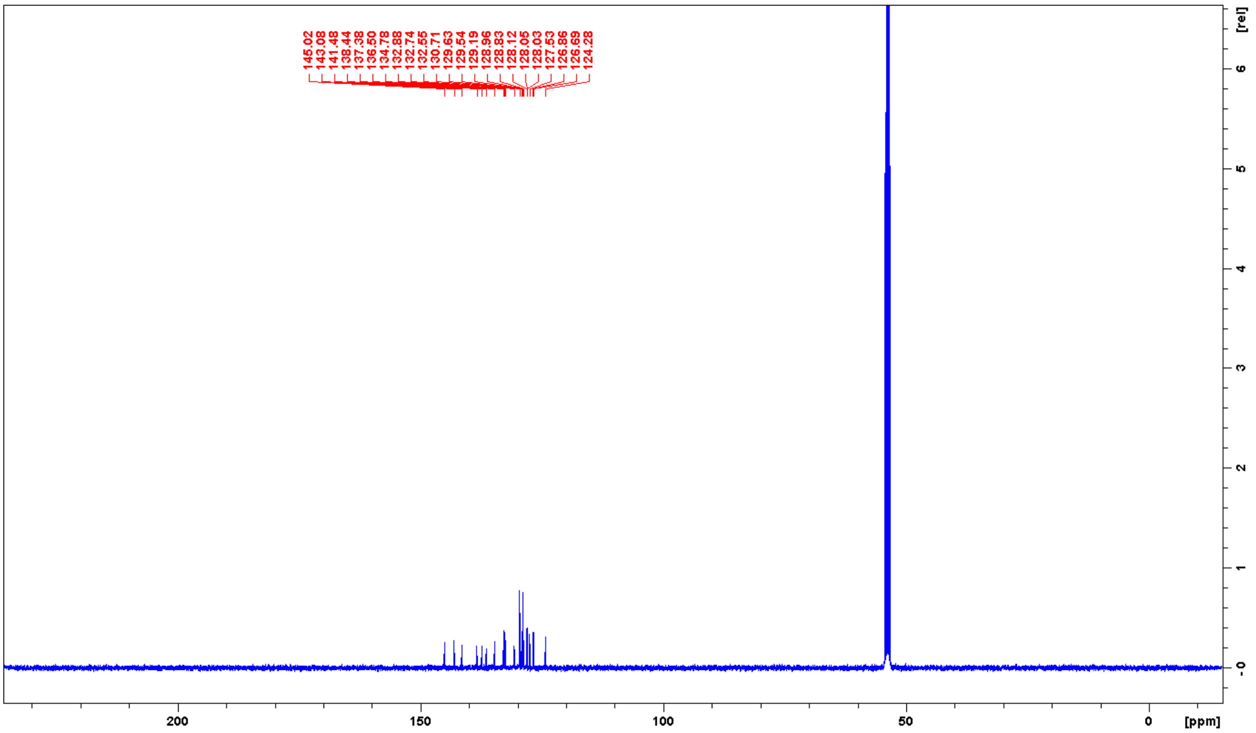

Figure S4. $^{13}C\{^{1}H\}$ NMR spectrum of **2** (101 MHz, CD_2Cl_2, 295 K).

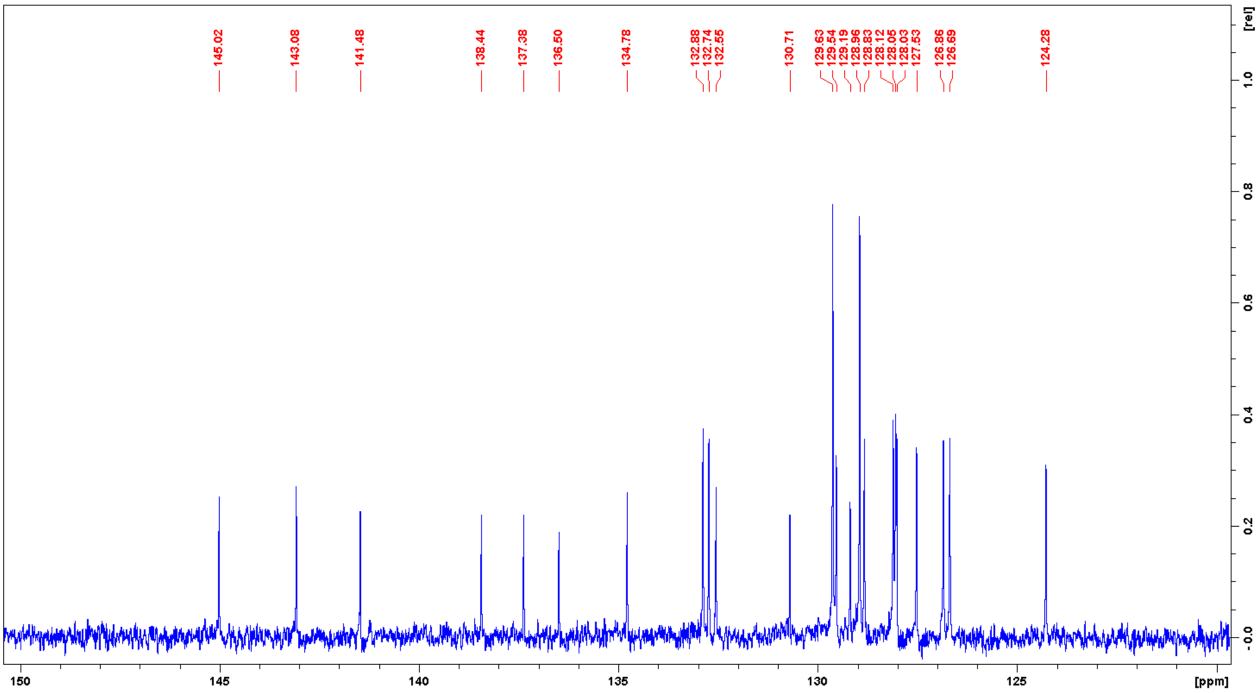

Figure S5. Magnified aromatic region of the $^{13}C\{^{1}H\}$ NMR spectrum of **2** (101 MHz, CD_2Cl_2, 295 K).

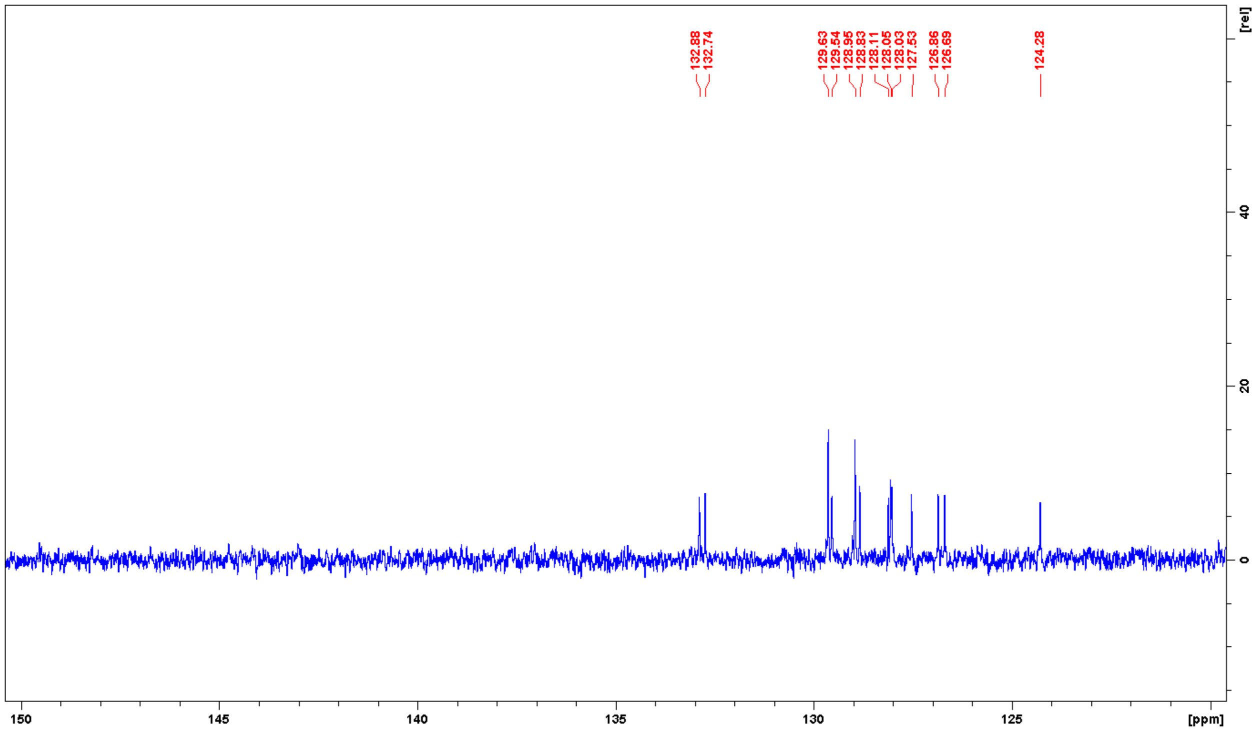

Figure S6. Magnified aromatic region of the DEPT-135 spectrum of **2** (101 MHz, CD_2Cl_2, 295 K).

4) X-ray Crystallography

Crystal data for Compound **2** ($C_{50}H_{30}$ • 0.189($C_4H_{10}O$) • 0.312(C_2H_3N)): *Mr* = 657.50, 0.093x0.060x0.058 mm^3, monoclinic space group $P2_1/c$, *a* = 10.8776(10) Å, α = 90°, *b* = 22.3515(19) Å, β = 96.779(5)° *c* = 30.055(3) Å, γ = 90°, *V* = 7256.1(11) $Å^3$, *Z* = 8, ρ(*calcd)* = 1.204 g·cm^{-3}, μ = 0.524 mm^{-1}, $F_{(000)}$ = 2758.0, *GooF*(F^2) = 1.034, R_1 = 0.0527, wR^2 = 0.1400 for I>2σ(I), R_1 = 0.0636, wR^2 = 0.1478 for all data, 14629 unique reflections [θ ≤ 72.534 °] with a completeness of 99.1 % and 994 parameters, 31 restraints.

5) Computations

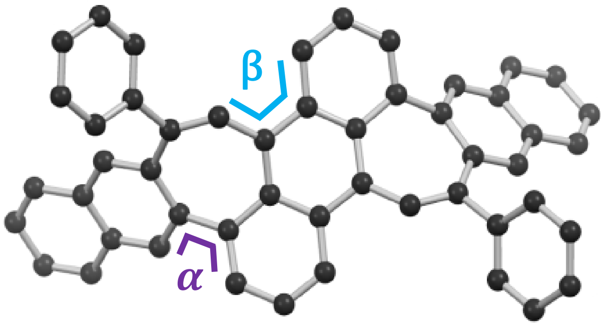

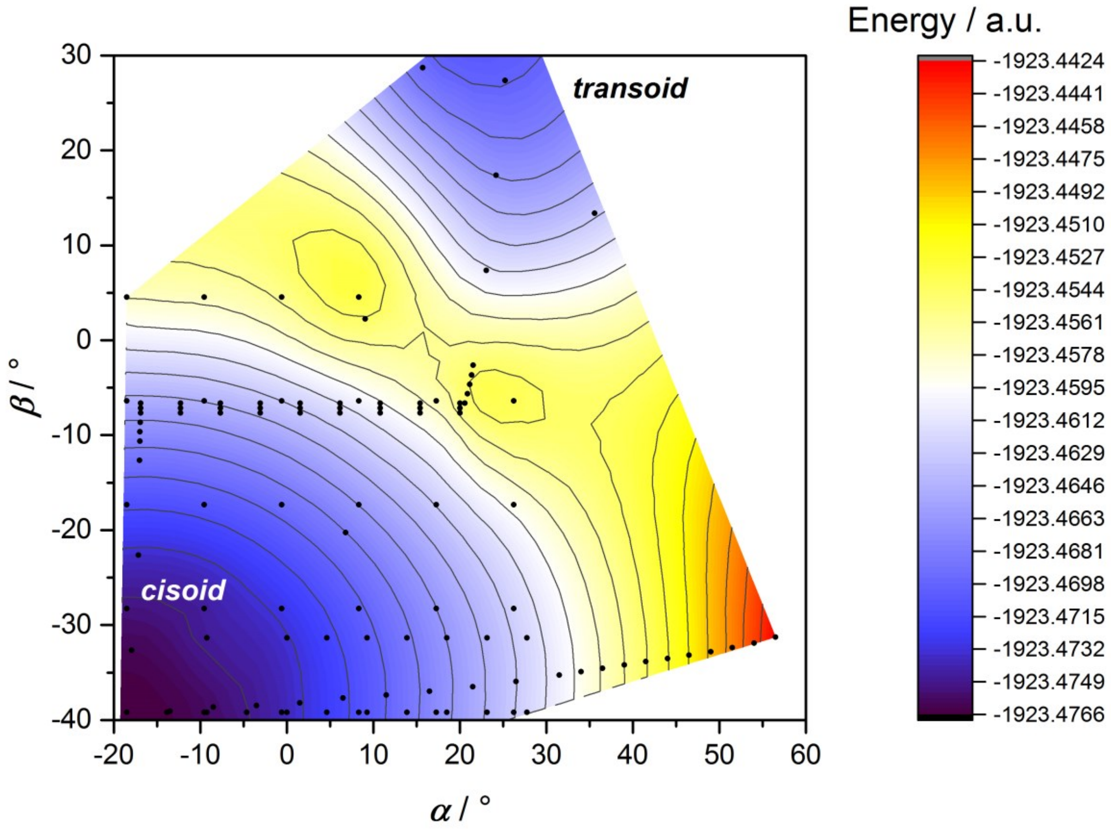

Figure S7. DFT-calculated electronic energies of **2** at various torsion angles *α* and *β* at the B3LYP/6-31+G* level of theory.

Table S2. Cartesian coordinates and absolute energy of the geometry-optimized structure (B3LYP/6-31+G*) of compound **2** in its cisoid conformation.

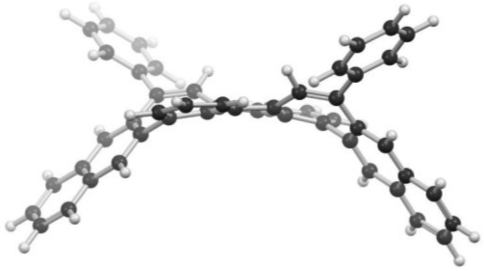

	x	y	z
C	–2.264587	0.064442	1.733886
C	–2.067317	0.207586	3.098028
H	–2.906407	0.061393	3.771698
C	–0.791016	0.478537	3.641488
H	–0.673616	0.565114	4.718636
C	0.304115	0.550245	2.817561
H	1.293480	0.642844	3.251282
C	0.168298	0.463158	1.398966
C	–1.157856	0.318775	0.840669
C	1.314916	0.536830	0.553597
C	1.157855	0.318182	–0.840948
C	–0.168292	0.462201	–1.399345
C	–1.314918	0.536481	–0.554027
C	2.264599	0.063172	–1.733965
C	2.067351	0.205276	–3.098208
H	2.906424	0.058491	–3.771776
C	0.791060	0.475894	–3.641873
H	0.673658	0.561693	–4.719084
C	–0.304072	0.548272	–2.818013
H	–1.293408	0.640602	–3.251860
C	–3.543242	–0.560471	1.284761
C	–4.320962	–0.124823	0.156091
C	–3.851579	0.919932	–0.773476
C	–2.546020	1.101383	–1.103506
H	–2.363815	1.854546	–1.866996
C	–4.016573	–1.627378	2.038809
H	–3.413696	–1.996896	2.863763
C	–5.246285	–2.278502	1.774479
C	–6.038523	–1.812266	0.679908
C	–5.541171	–0.741128	–0.098188
H	–6.135397	–0.403409	–0.942254
C	–5.718837	–3.370095	2.554239
H	–5.112617	–3.725177	3.384901
C	–6.926429	–3.968965	2.264516
H	–7.279509	–4.802527	2.866210
C	–7.715325	–3.503461	1.179920

H	–8.665571	–3.984370	0.962314
C	–7.280887	–2.449726	0.404772
H	–7.882122	–2.091951	–0.428224
C	–4.872676	1.769562	–1.459410
C	–4.856208	1.958279	–2.852201
H	–4.121488	1.428496	–3.453131
C	–5.785322	2.794572	–3.476611
H	–5.756725	2.918535	–4.556467
C	–6.753250	3.459209	–2.719724
H	–7.476685	4.110078	–3.204040
C	–6.787898	3.274130	–1.333602
H	–7.535455	3.787309	–0.733584
C	–5.863079	2.434124	–0.712550
H	–5.896708	2.298475	0.365265
C	3.543257	–0.561410	–1.284381
C	4.321050	–0.124857	–0.156103
C	3.851591	0.920438	0.772795
C	2.546028	1.102098	1.102692
H	2.363812	1.855723	1.865731
C	4.016560	–1.628868	–2.037653
H	3.413636	–1.999035	–2.862285
C	5.246337	–2.279721	–1.772955
C	6.038688	–1.812566	–0.678860
C	5.541348	–0.740874	0.098486
H	6.135640	–0.402495	0.942241
C	5.718846	–3.371912	–2.551900
H	5.112544	–3.727689	–3.382205
C	6.926509	–3.970486	–2.261854
H	7.279550	–4.804515	–2.862923
C	7.715522	–3.504065	–1.177740
H	8.665819	–3.984749	–0.959858
C	7.281128	–2.449726	–0.403384
H	7.882463	–2.091249	0.429239
C	4.872622	1.770569	1.458279
C	4.856241	1.959789	2.851002
H	4.121662	1.430065	3.452162
C	5.785246	2.796505	3.475008
H	5.756756	2.920868	4.554819
C	6.752950	3.461059	2.717764
H	7.476301	4.112262	3.201762
C	6.787480	3.275504	1.331704
H	7.534856	3.788646	0.731431
C	5.862775	2.435074	0.711052
H	5.896293	2.299045	–0.366724

E = –1923.47659054 a.u.

Table S3. Cartesian coordinates and absolute energy of the geometry-optimized structure (B3LYP/6-31+G*) of compound **2** in its transoid conformation.

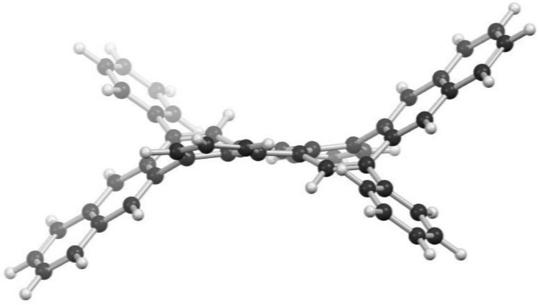

	x	y	z
H	–0.428557	3.372803	0.765671
C	0.417530	2.694468	0.757376
C	0.196046	1.313635	0.463797
C	2.782803	2.296991	0.970890
C	1.336875	0.444149	0.320110
C	1.685872	3.187785	0.927448
C	2.630999	0.937199	0.751180
H	1.848706	4.252587	1.072399
H	3.966657	1.246924	2.971011
H	3.764755	2.683905	1.225798
C	3.553138	–1.665750	–0.667951
C	4.223451	–1.115731	0.522349
C	5.328926	–1.790594	1.029458
C	3.741197	0.050750	1.212100
C	5.978602	–1.413648	2.227501
H	5.700857	–2.662850	0.500355
C	5.466746	–0.290606	2.947551
C	4.357890	0.405345	2.406797
C	4.376506	–2.427863	–1.656813
C	5.892364	–3.804898	–3.602262
C	5.539221	–1.856160	–2.205902
C	3.999545	–3.709605	–2.093333
C	4.748460	–4.391723	–3.055728
C	6.284515	–2.532952	–3.171918
H	5.850536	–0.867169	–1.879857
H	3.122537	–4.183794	–1.660177
H	4.440770	–5.385670	–3.371388
H	7.171564	–2.064816	–3.591693
H	6.475650	–4.333893	–4.351585
C	2.212150	–1.596905	–0.885451
C	1.138127	–0.841860	–0.246193
H	1.872100	–2.136184	–1.766323
H	0.428557	–3.372803	–0.765671
C	–0.417530	–2.694468	–0.757376
C	–0.196046	–1.313635	–0.463797

C	–2.782803	–2.296991	–0.970890
C	–1.336875	–0.444149	–0.320110
C	–1.685872	–3.187785	–0.927448
C	–2.630999	–0.937199	–0.751180
H	–1.848706	–4.252587	–1.072399
H	–3.966657	–1.246924	–2.971011
H	–3.764755	–2.683905	–1.225798
C	–3.553138	1.665750	0.667951
C	–4.223451	1.115731	–0.522349
C	–5.328926	1.790594	–1.029458
C	–3.741197	–0.050750	–1.212100
C	–5.978602	1.413648	–2.227501
H	–5.700857	2.662850	–0.500355
C	–5.466746	0.290606	–2.947551
C	–4.357890	–0.405345	–2.406797
C	–4.376506	2.427863	1.656813
C	–5.892364	3.804898	3.602262
C	–3.999545	3.709605	2.093333
C	–5.539221	1.856160	2.205902
C	–6.284515	2.532952	3.171918
C	–4.748460	4.391723	3.055728
H	–3.122537	4.183794	1.660177
H	–5.850536	0.867169	1.879857
H	–7.171564	2.064816	3.591693
H	–4.440770	5.385670	3.371388
H	–6.475650	4.333893	4.351585
C	–2.212150	1.596905	0.885451
C	–1.138127	0.841860	0.246193
H	–1.872100	2.136184	1.766323
C	7.098006	–2.119389	2.750587
H	7.485527	–2.973399	2.199356
H	8.534315	–2.274581	4.330824
C	7.679984	–1.729385	3.937850
H	7.634285	–0.321975	5.592423
C	7.167799	–0.617211	4.656069
H	5.690755	0.938802	4.719972
C	6.084895	0.085755	4.171708
C	–7.098006	2.119389	–2.750587
H	–7.485527	2.973399	–2.199356
H	–8.534315	2.274581	–4.330824
C	–7.679984	1.729385	–3.937850
H	–7.634285	0.321975	–5.592423
C	–7.167799	0.617211	–4.656069
H	–5.690755	–0.938802	–4.719972
C	–6.084895	–0.085755	–4.171708

E = –1923.47086100 a.u.

6) References

[S1] J. M. Farrell, D. Schmidt, V. Grande, F. Würthner, *Angew. Chem. Int. Ed.* **2017**, *56*, 11846-11850; *Angew. Chem.* **2017**, *129*, 12008-12012.
[S2] S. S. Zalesskiy, V. P. Ananikov, *Organometallics* **2012**, *31*, 2302-2309.
[S3] G. Seybold, G. Wagenblast, *Dyes Pigm.* **1989**, *11*, 303-317.
[S4] A. J. Fry, *Laboratory Techniques in Electroanalytical Chemistry*, 2nd ed., Marcel Dekker Ltd, New York, **1996**.
[S5] G. Sheldrick, *Acta Crystallogr. A* **2008**, *64*, 112-122.
[S6] Becke, A. D. *Phys. Rev. A* **1988**, *38*, 3098-3100.
[S7] Lee, C.; Yang, W.; Parr, R. G. *Phys. Rev. B* **1988**, *37*, 785-789.
[S8] Becke, A. D. *J. Chem. Phys.* **1993**, *98*, 5648-5652.
[S9] Krishnan, R.; Binkley, J. S.; Seeger, R.; Pople, J. A. *J. Chem. Phys.* **1980**, *72*, 650-654.
[S10] Clark, T.; Chandrasekhar, J.; Spitznagel, G. W.; Schleyer, P. v. R. *J. Comput. Chem.* **1983**, *4*, 294-301.
[S11] Frisch, M. J.; Pople, J. A.; Binkley, J. S. *J. Chem. Phys.* **1984**, *80*, 3265-3269.
[S12] Gaussian 09, Revision D.01, Frisch, M. J.; Trucks, G. W.; Schlegel, H. B.; Scuseria, G. E.; Robb, M. A.; Cheeseman, J. R.; Scalmani, G.; Barone, V.; Mennucci, B.; Petersson, G. A.; Nakatsuji, H.; Caricato, M.; Li, X.; Hratchian, H. P.; Izmaylov, A. F.; Bloino, J.; Zheng, G.; Sonnenberg, J. L.; Hada, M.; Ehara, M.; Toyota, K.; Fukuda, R.; Hasegawa, J.; Ishida, M.; Nakajima, T.; Honda, Y.; Kitao, O.; Nakai, H.; Vreven, T.; Montgomery, J. A.; Peralta, J. E.; Ogliaro, F.; Bearpark, M.; Heyd, J. J.; Brothers, E.; Kudin, K. N.; Staroverov, V. N.; Keith, T.; Kobayashi, R.; Normand, J.; Raghavachari, K.; Rendell, A.; Burant, J. C.; Iyengar, S. S.; Tomasi, J.; Cossi, M.; Rega, N.; Millam, J. M.; Klene, M.; Knox, J. E.; Cross, J. B.; Bakken, V.; Adamo, C.; Jaramillo, J.; Gomperts, R.; Stratmann, R. E.; Yazyev, O.; Austin, A. J.; Cammi, R.; Pomelli, C.; Ochterski, J. W.; Martin, R. L.; Morokuma, K.; Zakrzewski, V. G.; Voth, G. A.; Salvador, P.; Dannenberg, J. J.; Dapprich, S.; Daniels, A. D.; Farkas, O.; Foresman, J. B.; Ortiz, J. V.; Cioslowski, J.; Fox, D. J. Gaussian, Inc., Wallingford CT, 2013.
